# The MDM2-p53 Axis in Osteosarcoma: Current Understanding of Regulatory Mechanisms and Targeted Therapeutic Strategies

**DOI:** 10.3390/ph19030476

**Published:** 2026-03-13

**Authors:** Wenxia Deng, Songyan Gao, Lige Yan, Qiuju Su, Si Chen

**Affiliations:** 1 School of Medicine, Shanghai University, Shanghai 200444, China; dengwenxia@shu.edu.cn (W.D.); ylg1130@shu.edu.cn (L.Y.); 2 Institute of Translational Medicine, Shanghai University, Shanghai 200444, China; sy_gaosmmu@163.com; 3 Department of Rehabilitation Medicine, The Affiliated Wuxi People’s Hospital of Nanjing Medical University, Wuxi People’s Hospital, Wuxi Medical Center, Nanjing Medical University, Wuxi 214023, China

**Keywords:** osteosarcoma, MDM2-p53, targeted therapy, drug resistance

## Abstract

Osteosarcoma, the most prevalent primary malignant bone tumor in children and adolescents, is characterized by high rates of metastasis, recurrence, and chemotherapy resistance, leading to suboptimal patient survival. The MDM2-p53 pathway plays a pivotal role in its tumorigenesis and progression, where dysregulation leads to loss of p53 function. This review systematically elucidates the molecular mechanisms of this pathway and summarizes diverse targeted therapeutic strategies, including small-molecule MDM2 inhibitors, mutant p53 reactivators, and innovative modalities such as gene therapy and Proteolysis Targeting Chimeras (PROTACs). Despite demonstrating potent preclinical activity with low IC_50_ values, the clinical translation of these agents has faced significant challenges. Early-generation MDM2 inhibitors (e.g., RG7112, Idasanutlin) showed limited monotherapy efficacy and dose-limiting toxicities like thrombocytopenia, halting their development at early-phase clinical trials. In contrast, novel MDM2 inhibitors like APG-115 have advanced to Phase II trials, marking a significant breakthrough. Although not yet tested in dedicated osteosarcoma cohorts, their safety and efficacy in MDM2-amplified solid tumors provide a critical foundation for the development of precision medicine and combination regimens for osteosarcoma. Future efforts to accelerate drug development may leverage single-cell sequencing and AI-aided drug design to decipher osteosarcoma heterogeneity and optimize drug profiles for reduced toxicity.

## 1. Introduction

Osteosarcoma (OS) is one of the most prevalent primary malignant bone tumors, with a particularly high incidence in children and adolescents [1]. It is characterized by high local aggressiveness, a propensity for distant metastasis, and a significant risk of recurrence, predominantly affecting the metaphysis of long bones [2]. While current therapeutic modalities, including surgery and chemotherapy, have improved clinical outcomes, the prognosis remains poor due to the tumor’s intrinsic drug resistance and high recurrence rates [3]. The limited efficacy of chemotherapy in many cases results in a five-year survival rate of only 20–30% for patients with metastatic or recurrent disease, a figure significantly lower than that of many other malignancies [4]. Furthermore, novel immunotherapies, such as immune checkpoint inhibitors, have demonstrated limited efficacy in osteosarcoma and have yet to achieve widespread clinical application [5]. This underscores a critical challenge in the field: the lack of effective and precise therapeutic targets. Therefore, identifying novel molecular drivers of osteosarcoma initiation and progression, and developing corresponding targeted therapies, is an urgent imperative.

The p53 protein is a pivotal tumor suppressor regulating cell cycle arrest, DNA repair, apoptosis, and senescence. Since its discovery in the 1970s, p53 dysfunction has been closely linked to the pathogenesis of numerous cancers [6,7]. Under physiological conditions, p53 is activated in response to cellular stress, such as DNA damage. By transactivating downstream target genes, it induces cell cycle arrest, apoptosis, senescence, or autophagy, thereby eliminating damaged cells and suppressing tumorigenesis [8]. Murine double minute 2 (MDM2) is a key endogenous negative regulator of p53 [9]. MDM2 expression is activated by p53, forming a critical negative feedback loop. The MDM2 protein then binds to p53’s transactivation domain, inhibiting its transcriptional activity and promoting its polyubiquitination and proteasomal degradation [10]. This autoregulatory circuit maintains p53 at low basal levels in unstressed cells, preventing potential damage from its overactivation and ensuring cellular homeostasis.

Approximately 50% of human cancers harbor mutations in the *TP53* gene. Mutant p53 proteins often lose wild-type transcriptional activity and, consequently, the ability to induce MDM2 expression [11,12]. This disrupts the negative feedback loop, leading to the accumulation of mutant p53 in tumor cells and contributing to oncogenesis [13]. Additionally, MDM2 itself is frequently overexpressed in cancers through gene amplification or other mechanisms. Elevated MDM2 potently inhibits wild-type p53 function, further driving tumor progression [14]. In osteosarcoma, disruption of the MDM2-p53 axis is a common event. This dysregulation not only leads to aberrant accumulation of the osteogenic master regulator Runx2, promoting the malignant transformation of osteoblasts [15], but also fosters tumor growth and chemoresistance through mechanisms like metabolic reprogramming [16].

Consequently, therapeutic strategies aimed at disrupting the MDM2-p53 interaction or reactivating mutant p53 have long been pursued. From early small-molecule MDM2 inhibitors like Nutlins [17] to the mutant p53 reactivator APR-246 [18], and more recently, PROTACs designed to degrade mutant p53 or MDM2 [19], continuous advancements have offered new hope. However, challenges related to hematological toxicity and acquired resistance have emerged during clinical translation, highlighting the need for further optimization. This article systematically reviews the core mechanisms of the MDM2-p53 axis in osteosarcoma pathogenesis and classifies therapeutic agents targeting this axis, aiming to provide a theoretical foundation and future directions for the precision treatment of osteosarcoma.

## 2. Molecular Mechanisms of the MDM2-p53 Pathway

**Figure 1:**
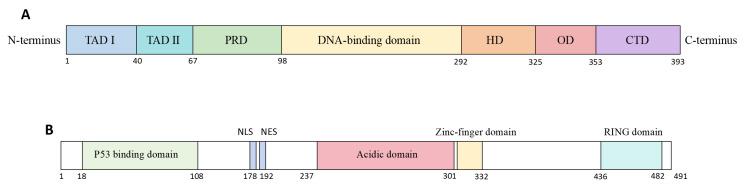
Structural organization of p53α and MDM2 proteins. (**A**) Schematic diagram of p53α structural domains. Key domains include: Transactivation Domain I (TAD I); Transactivation Domain II (TAD II); Proline-Rich Domain (PRD); DNA-Binding Domain (DBD); Hinge Domain (HD); Oligomerization Domain (OD); and C-Terminal Domain (CTD). (**B**) Schematic diagram of MDM2 protein domains. Key domains include: p53-Binding Domain; Nuclear Localization Signal (NLS); Nuclear Export Signal (NES); Acidic Domain; Zinc Finger Domain; and RING Domain.

As the master regulator of the cellular damage response, p53 is encoded by the *TP53* gene located on chromosome 17p13.1 [20]. The canonical p53α isoform comprises 393 amino acids [21] and as schematically depicted in Figure 1A, it contains seven core functional domains: the N-terminal transactivation domains (TAD I/II) responsible for initiating transcription of downstream target genes upon binding to their partner proteins [22]; the proline-rich domain (PRD) mediating protein–protein interactions [23]; the central DNA-binding domain (DBD) which, assisted by the C-terminal oligomerization domain (OD), recognizes specific p53 response elements [24]; and the C-terminal regulatory domain (CTD) with additional DNA-binding and regulatory functions [25].

MDM2 serves as the primary negative regulator of p53, predominantly by promoting its ubiquitination and proteasomal degradation [26]. As illustrated in Figure 1B, the structural organization of MDM2 features an N-terminal hydrophobic pocket that binds the p53 TAD, directly blocking transcriptional activity [27]. Central nuclear localization and export signals (NLS/NES) enable MDM2 to shuttle p53 out of the nucleus [28], while its acidic domain facilitates degradation through both ubiquitin-dependent and -independent mechanisms [29]. The zinc finger domain can be bound by ribosomal proteins under stress, inhibiting MDM2’s E3 ligase function [30]. Finally, the C-terminal RING domain confers E3 ubiquitin ligase activity essential for p53 ubiquitination and MDM2 dimerization [31] (Figure 1B).

**Figure 2:**
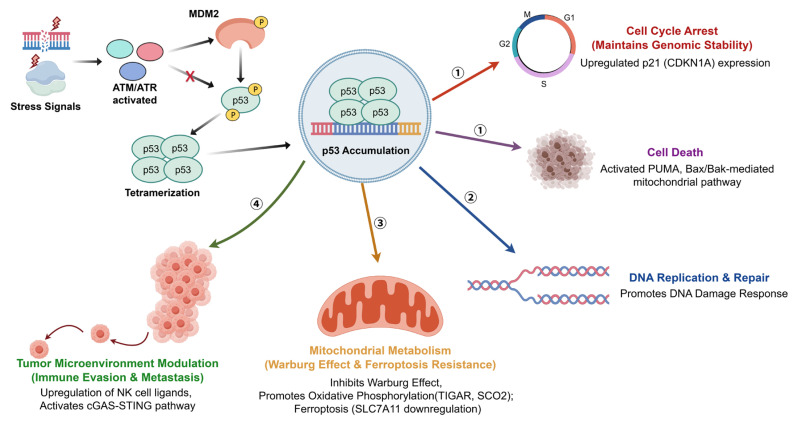
Activation of the MDM2-p53 pathway under stress conditions and its multidimensional downstream regulatory network. Upon exposure to stress signals such as DNA damage, kinases including ATM/ATR are activated, leading to the specific phosphorylation of MDM2 and p53; this disrupts their interaction, allowing p53 to rapidly accumulate and tetramerize into an active conformation. The accumulated p53 functions as a master transcription factor to execute tumor suppression via multiple pathways: ① Cell cycle arrest and apoptosis: It upregulates p21 (CDKN1A) to enforce cell cycle arrest and maintain genomic stability, and activates the PUMA and Bax/Bak-mediated mitochondrial pathway to trigger cell death. ② DNA replication and repair: It promotes the DNA damage response. ③ Metabolic reprogramming: It inhibits the Warburg effect and promotes oxidative phosphorylation via TIGAR and SCO2, while inducing ferroptosis by downregulating SLC7A11. ④ Tumor microenvironment modulation: It counteracts immune evasion and metastasis by upregulating NK cell ligands and activating the cGAS-STING pathway via TREX1 downregulation. Conversely, during normal skeletal development, hyper-suppression of p53 by MDM2 leads to aberrant upregulation of Runx2, which blocks the normal differentiation of mesenchymal stem cells into osteoblasts and drives osteosarcoma tumorigenesis. (Created by Figdraw, accessed on 26 February 2026).

MDM2 and p53 engage in a precise autoregulatory negative feedback loop. Under basal conditions, MDM2 binds via its N-terminal pocket to a critical α-helix (involving Phe19, Trp23, and Leu26) within the p53 TAD, occluding its transactivation surface [32,33]. The level of MDM2 dictates the outcome: low levels induce p53 mono-ubiquitination and nuclear export [34], whereas high levels drive its poly-ubiquitination and proteasomal degradation, maintaining low steady-state p53 levels [35]. Cellular stressors such as DNA damage activate kinases like ATM and ATR, which phosphorylate both proteins, disrupting their interaction [36]. This leads to p53 stabilization, tetramerization, and activation of downstream target genes governing cell fate [37]. As depicted in Figure 2, the regulatory network of the MDM2-p53 axis involves diverse physiological processes in addition to canonical tumor suppression. In skeletal development, MDM2-mediated inhibition of p53 is essential for osteogenic differentiation of mesenchymal stem cells [15], and its dysregulation can contribute to osteosarcomagenesis [38]. Metabolically, p53 can suppress the Warburg effect by upregulating TIGAR and SCO2 to promote oxidative phosphorylation [39], and induce ferroptosis by repressing SLC7A11 [40]. In immune surveillance, p53 enhances tumor cell recognition by upregulating ligands for Natural Killer (NK) cells [41] and activates the cGAS-STING pathway to elicit a type I interferon response, further inhibiting tumor progression [42].

## 3. The MDM2-p53 Pathway in Osteosarcoma

Functional dysregulation of the MDM2-p53 pathway is a core molecular event driving the initiation and progression of osteosarcoma. Genome-wide studies indicate that the functional inactivation of this regulatory axis primarily occurs via two major, often mutually exclusive, genetic mechanisms: (1) structural and functional aberrations of the *TP53* gene, directly compromising the tumor-suppressive activity of the p53 protein; and (2) genomic amplification of MDM2, leading to excessive inhibition of wild-type p53 function. The specific mechanism underlying this imbalance dictates the subsequent selection of targeted therapeutic strategies for osteosarcoma.

### 3.1. TP53 Gene Mutations

Osteosarcoma is intrinsically linked to abnormalities in the tumor suppressor gene *TP53*. The p53 protein, often termed the “guardian of the genome,” functions as a tetrameric transcription factor that binds to specific p53 response elements (REs) on DNA, regulating critical processes such as cell cycle arrest, DNA repair, apoptosis, and senescence to ensure cellular homeostasis [43]. The *TP53* gene is crucial for maintaining bone homeostasis. Li-Fraumeni syndrome (LFS), a hereditary cancer predisposition caused by germline *TP53* mutations, confers a high lifetime risk of various malignancies, with approximately 50% of carriers developing cancers—including sarcomas, brain tumors, and breast cancer—before age 30. Osteosarcoma is one of the most frequently observed tumors in LFS patients [44]. Beyond hereditary factors, somatic *TP53* mutations or structural variations are present in over 50% of sporadic osteosarcoma cases [45]. Unlike many tumor suppressor genes (e.g., RB1) commonly inactivated by deletions or truncations, the majority of *TP53* alterations in osteosarcoma are missense mutations concentrated in the DNA-binding domain (DBD) [46]. This distinct pattern leads to the expression of mutant p53 (mutp53) proteins that not only lose their normal transcriptional activity but can also exert dominant-negative effects over wild-type p53 and acquire novel oncogenic functions [47].

In osteosarcoma, *TP53* missense mutations have been identified at over 190 codons, with hotspots at R175, G245, R248, R249, R273, and R282 [48]. Based on molecular mechanism, these hotspot mutations fall into two main classes: (1) Contact mutations (e.g., R248, R273), which directly disrupt specific hydrogen bonds and salt bridges between p53 and the DNA major groove, abolishing sequence-specific DNA binding [49]; and (2) Conformational (or structural) mutations (e.g., R175H), which destabilize the DBD’s hydrophobic core, inducing local or global unfolding. This conformational change not only inactivates p53 but also exposes hydrophobic regions, promoting mutp53 misfolding, aggregation, and proteotoxic stress [50,51].

**Figure 3:**
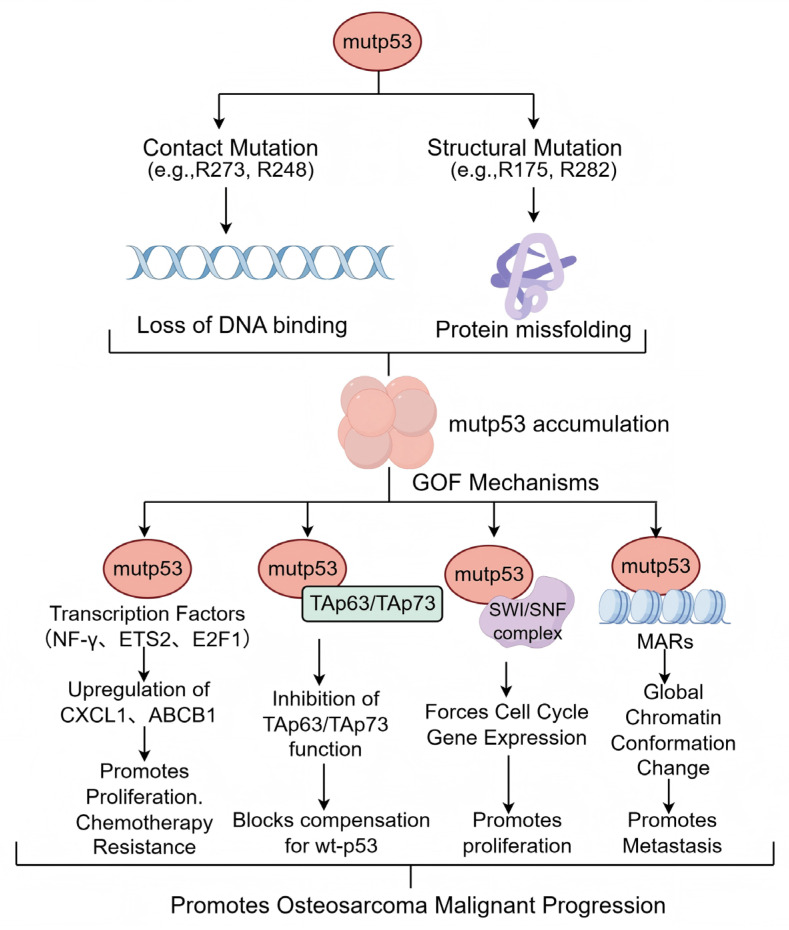
Mechanisms by which functional p53 mutations promote malignant progression in osteosarcoma. TP53 mutations drive the malignant progression of osteosarcoma through distinct pathways. Mutant p53 (mutp53) is categorized into contact mutations and structural mutations, which result in the loss of DNA-binding capacity and protein misfolding, respectively. These alterations lead to the stabilization and substantial accumulation of mutp53 within tumor cells. The accumulated protein then exerts oncogenic gain-of-function (GOF) effects through multiple mechanisms. First, mutp53 interacts with various transcription factors—such as NF-Y, ETS2, and E2F1—to promote cell proliferation and induce chemotherapy resistance. It also binds to and suppresses the tumor-suppressive activities of p53 family members, TAp63 and TAp73, thereby abrogating growth inhibition. Concurrently, through interaction with the SWI/SNF complex, mutp53 sustains signaling pathways that drive abnormal tumor cell proliferation. Furthermore, mutp53 specifically recognizes and binds to Matrix Attachment Regions (MARs), inducing global changes in chromatin spatial conformation; this empowers osteosarcoma cells to detach from the primary site and initiate distant metastasis. (Created by Figdraw, accessed on 26 February 2026).

The concept of mutant p53 gain-of-function (GOF) emerged over two decades ago. Substantial experimental evidence now confirms that accumulated mutp53 can acquire oncogenic properties distinct from wild-type p53, promoting osteosarcoma malignancy [52]. These GOF activities include enhancing tumor cell proliferation, survival, migration, invasion, chemoresistance, and metabolic reprogramming. Although mutp53 loses its canonical transcriptional function, it modulates gene expression through alternative mechanisms [12]. A schematic representation of the major GOF mechanisms of mutp53, which will be discussed below, is shown in Figure 3. First, mutp53 can interact with transcription factors such as NF-Y, ETS2, E2F1, and VDR via protein–protein interactions, upregulating genes like *CXCL1* and *ABCB1* to promote proliferation and chemoresistance [53,54,55]. Second, mutp53 can bind to and inhibit the transcriptional activity of p53 family members TAp63 and TAp73, blocking their compensatory tumor-suppressive effects [56]. Furthermore, mutp53 can interact with chromatin remodelers like the SWI/SNF complex to aberrantly activate cell cycle drivers [57]. Crucially, mutp53 can bind to matrix attachment regions (MARs), inducing global chromatin conformational changes that may facilitate tumor cell detachment and metastasis [58].

### 3.2. Aberrant Expression and Regulation of MDM2/MDM4

MDM2 is a critical negative regulator of p53, controlling its transcriptional activity, stability, and subcellular localization. Aberrant MDM2 overexpression represents another core mechanism leading to wild-type p53 functional loss. Approximately 16% of osteosarcomas exhibit *MDM2* gene amplification or protein overexpression, which abrogates p53-dependent functions like apoptosis and cell cycle arrest [59].

**Figure 4:**
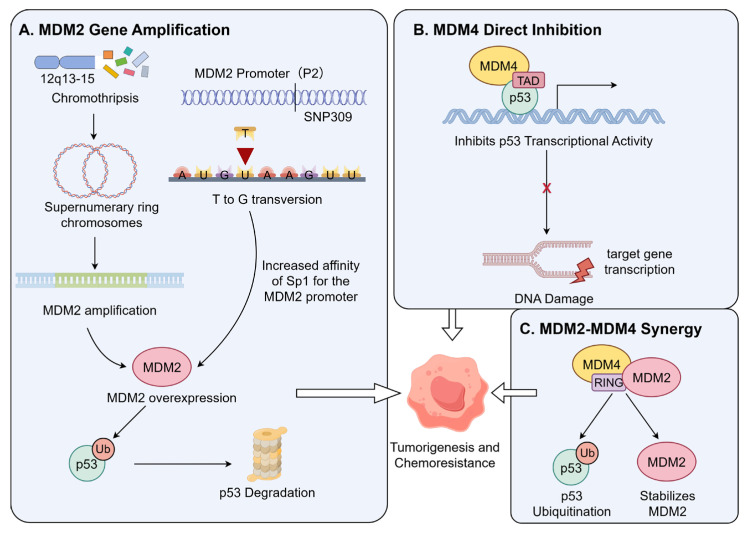
Mechanisms of MDM2/MDM4-mediated p53 inactivation in osteosarcoma. (**A**) MDM2 blocks p53 function through two primary mechanisms involving gene amplification and overexpression. First, chromothripsis in the 12q13-15 region leads to the formation of *MDM2*-containing supernumerary ring chromosomes, causing a dramatic increase in gene copy number and protein overexpression. Second, a Single Nucleotide Polymorphism (*SNP309* T>G) in the *MDM2* promoter region increases the affinity for the transcription factor Sp1, thereby enhancing *gene* transcription; both pathways utilize the E3 ubiquitin ligase activity of MDM2 to mediate proteasomal degradation of p53. (**B**) MDM4 primarily exerts direct inhibition by binding to the p53 Transactivation Domain (TAD) via its N-terminal domain, directly blocking the transcriptional activation of downstream target genes. (**C**) MDM4 and MDM2 exhibit a synergistic effect by forming a heterodimer through their RING domains. This complex not only prevents MDM2 auto-ubiquitination, thereby increasing its stability, but also exhibits stronger E3 ligase activity than the MDM2 monomer, accelerating p53 ubiquitination and degradation, which ultimately promotes tumorigenesis and leads to chemotherapy resistance. (Created by Figdraw, accessed on 26 February 2026).

Gene amplification is the primary mechanism by which MDM2 blocks p53 function and shows significant subtype specificity. *MDM2* amplification is a characteristic genetic hallmark of low-grade parosteal osteosarcoma and well-differentiated liposarcoma, but is relatively rare in conventional high-grade osteosarcoma [60]. This amplification is frequently associated with chromothripsis in the chromosomal 12q13-15 region, leading to the formation of *MDM2*-containing supernumerary ring or giant marker chromosomes. These structures can increase *MDM2* copy number by tens to hundreds of folds, resulting in high intracellular MDM2 protein levels that continuously ubiquitinate and degrade wild-type p53 [61]. Genomic analyses confirm a significant mutual exclusivity between *MDM2* amplification and *TP53* mutations, suggesting that MDM2 amplification alone is sufficient to inactivate the p53 pathway during tumorigenesis [62] (Figure 4A).

In conventional osteosarcomas lacking gene amplification, MDM2 protein overexpression is more common, often driven by single nucleotide polymorphisms (SNPs) affecting transcription. The most studied is *SNP309* (T → G) in the *MDM2P2* promoter region, which creates a high-affinity binding site for the transcription factor Sp1, significantly enhancing basal *MDM2* transcription and protein levels [63,64]. Clinically, the SNP309 G allele is associated with elevated MDM2 levels, attenuated p53 pathway response to DNA damage, earlier sarcoma onset, and reduced chemotherapy sensitivity [63,65] (Figure 4A).

Beyond MDM2, its structural homolog MDM4 (MDMX) is also a key negative regulator of p53. The specific molecular mechanism underlying the independent inhibitory role of MDM4 is detailed in Figure 4B. Unlike MDM2, MDM4 primarily inhibits p53’s transcriptional activity without directly promoting its degradation. The MDM4 protein binds to the p53 transactivation domain (TAD), blocking its interaction with the transcriptional machinery [66]. In addition to this independent function, the synergistic interplay between MDM4 and MDM2 is further elaborated in Figure 4C. MDM4 can heterodimerize with MDM2 via their RING domains; this interaction stabilizes MDM2 by inhibiting its auto-ubiquitination, thereby enhancing MDM2-mediated p53 ubiquitination and degradation [67]. Furthermore, the MDM2-MDM4 heterodimer exhibits stronger E3 ubiquitin ligase activity toward p53 than the MDM2 homodimer [68]. Thus, MDM4 overexpression can both directly inhibit p53 transcription and indirectly accelerate p53 clearance by stabilizing MDM2 [69].

### 3.3. Non-Coding RNA Regulatory Networks

Non-coding RNAs (ncRNAs), including microRNAs (miRNAs), long non-coding RNAs (lncRNAs), and circular RNAs (circRNAs), are transcribed from the genome but not translated into protein [70]. Advances in transcriptomics have revealed that aberrant ncRNA expression is intimately associated with tumorigenesis, progression, and therapy resistance in various cancers, including osteosarcoma [71,72].

**Figure 5:**
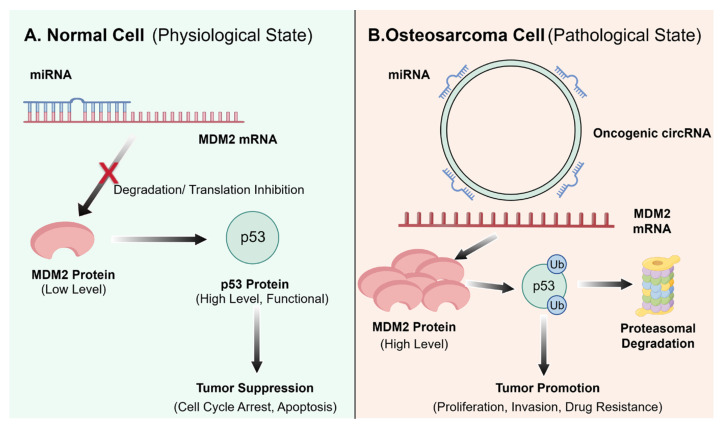
Mechanism of circular RNA-mediated regulation of MDM2 protein levels in osteosarcoma. (**A**) In the normal physiological state, specific miRNAs exert negative regulation by binding to *MDM2 mRNA*, inducing either mRNA degradation or translational inhibition; this maintains MDM2 protein expression at low levels, thereby ensuring p53 stability and its tumor-suppressive function. (**B**) In the pathological state of osteosarcoma, oncogenic circRNAs are upregulated and competitively bind to these inhibitory miRNAs, effectively relieving their suppression of *MDM2* mRNA. This leads to the aberrant accumulation of MDM2 protein, which subsequently accelerates ubiquitin-mediated proteasomal degradation of p53, ultimately driving tumor proliferation, invasion, and the acquisition of drug resistance. (Created by Figdraw, accessed on 26 February 2026).

Among ncRNAs, circRNAs have garnered significant interest in MDM2-p53 pathway regulation due to their unique closed-loop structure, which confers high stability and resistance to exonuclease degradation, allowing for sustained regulatory functions within cells [73]. As illustrated in Figure 5A, under physiological conditions, specific miRNAs (e.g., *miR-133b*, *miR-135a*) bind to and degrade MDM2 mRNA, ensuring that p53 is maintained at appropriate levels [74]. Conversely, Figure 5B delineates how this balance is disrupted in osteosarcoma, certain oncogenic circRNAs can act as competitive endogenous RNAs (ceRNAs). They sequester miRNAs that normally target MDM2 mRNA, relieving this post-transcriptional repression. This leads to aberrant MDM2 protein accumulation, accelerated p53 ubiquitination and degradation, and ultimately promotes tumor proliferation, invasion, and drug resistance [75,76]. For instance, *circ_0102049* has been shown to sponge *miR-1304-5p*, a negative regulator of MDM2, resulting in elevated MDM2 levels, p53 degradation, and enhanced osteosarcoma malignancy. Preclinical studies suggest that silencing such oncogenic circRNAs through the use of siRNA can restore p53 activity and inhibit tumor growth, presenting a potential strategy to overcome resistance [77]. However, the development of effective RNA-based therapeutics for osteosarcoma remains at the preclinical stage, hindered by challenges in delivery and stability.

## 4. Therapeutic Strategies Targeting the MDM2-p53 Pathway

### 4.1. Monotherapy Strategies

#### 4.1.1. Small-Molecule MDM2 Inhibitors

Given the prevalence of MDM2 overexpression leading to wild-type p53 inactivation in osteosarcoma, restoring p53 activity using MDM2 antagonists has emerged as a promising therapeutic strategy. Table 1 summarizes the characteristics and current clinical status of several developed small-molecule MDM2 inhibitors, with notable candidates including Nutlin-3a, RG7112, RG7388, AMG-232, and APG-115 advancing to clinical trials [78] (Table 1).

The Nutlin series, represented by Nutlin-3a, are the first class of highly specific, non-peptide small-molecule MDM2 antagonists. They bind to MDM2’s p53-binding pocket, preventing p53-MDM2 interaction, stabilizing p53, and inducing p53-dependent cell cycle arrest and apoptosis [17,99]. Nutlin-3a has shown anti-tumor effects in osteosarcoma by inducing G1/G2 phase arrest and apoptosis [100]. However, its poor metabolic stability and low oral bioavailability [101] spurred the development of second-generation compounds [81]. Optimized derivatives like RG7112 and RG7388 demonstrated tumor growth inhibition and apoptosis induction in vitro and in xenograft models [82]; however, RG7112 failed in further development due to severe hematological toxicity [102]. Recent clinical studies in pediatric/adolescent solid tumors showed RG7388 had suboptimal monotherapy efficacy coupled with significant myelosuppression and gastrointestinal toxicity, leading to the termination of its global development in 2024 [84].

To address the metabolic and tolerability limitations of earlier drugs, AMG-232 was developed as a more promising oral MDM2 inhibitor. It binds with high affinity to the Phe19, Trp23, and Leu26 residues on MDM2, potently stabilizing and activating p53 [103]. Compared to Nutlins, AMG-232 showed significant anti-tumor activity in the SJSA-1 osteosarcoma model without obvious toxicity [104]. A Phase I trial reported good tolerability in patients with advanced *TP53* wild-type solid tumors and multiple myeloma, providing a dosage rationale for subsequent trials in sarcomas [86]. AMG-232 also shows synergistic potential in combination with MEK inhibitors or chemotherapeutics, enhancing cytotoxicity against tumor cells [105,106]. Its research focus has thus shifted from monotherapy to combination regimens for specific molecular subtypes [107].

The concept of combination therapy is further exemplified by APG-115. Preclinical studies indicate that p53 pathway activation can upregulate tumor cell PD-L1 expression and modulate the tumor microenvironment (TME) by promoting M1 macrophage polarization and increasing CD8+ T cell infiltration, suggesting MDM2 inhibitors may overcome immunotherapy resistance [108]. In advanced *TP53*wild-type, *MDM2*-amplified solid tumors, APG-115 demonstrated a favorable safety profile and anti-tumor activity, achieving high disease control rates and prolonged tumor stabilization in some patients [92]. These findings support its clinical exploration in osteosarcoma with similar pathogenesis. APG-115 also enhances the efficacy of anti-PD-1 antibodies by activating p53 within immune cells [109]. This strategic shift toward immune-synergistic therapy offers a new direction to address the limited efficacy and toxicity of MDM2 inhibitor monotherapy.

While the above inhibitors have advanced *p53* activation and immune synergy, most exhibit high affinity only for MDM2. Inhibition of MDM2 can lead to compensatory binding of its homolog MDM4 to p53, sustaining p53 suppression and fostering resistance. To overcome this, Sulanemadlin (ALRN-6924), a first-in-class stapled peptide, was developed to simultaneously bind and inhibit both MDM2 and MDM4, fully releasing wild-type p53 from dual inhibition [98]. Early attempts to use it as a chemoprotectant in *p53*-mutant tumors were halted due to severe hematological adverse events in a Phase I trial (https://clinicaltrials.gov/study/NCT05622058, accessed on 26 February 2026) Current clinical research refocuses on its therapeutic potential as a dual MDM2/MDMX inhibitor. A Phase I/II trial in patients with *TP53*wild-type advanced solid tumors or lymphomas reported a favorable safety profile with minimal myelosuppressive toxicity and preliminary anti-tumor activity [110]. Subsequently, ALRN-6924 combined with CDK4/6 inhibitors showed robust synergistic anti-proliferative effects (https://clinicaltrials.gov/study/NCT02264613, accessed on 26 February 2026). Its unique dual-target mechanism and favorable bone marrow tolerability may provide a novel, low-toxicity paradigm for osteosarcoma targeted therapy.

#### 4.1.2. Functional Reactivation of Mutant p53

High-frequency *TP53* mutations are prevalent in osteosarcoma. While restoring mutant p53 (mutp53) function has long been proposed as a therapeutic strategy, its “undruggable” nature—lack of well-defined binding pockets—has hindered drug development [111]. Nonetheless, as detailed in Table 2, scientific advances have identified compounds capable of reactivating mutp53, such as APR-246 and Arsenic Trioxide (ATO) [112].

APR-246 is the most clinically advanced mutp53-targeted therapy. It is a prodrug converted to its active metabolite, methylene quinuclidinone (MQ), in acidic cellular environments. MQ specifically binds to cysteine residues (Cys124 and Cys277) in the mutp53 core domain, restoring its wild-type conformation and transcriptional activity [126]. Research on APR-246 in osteosarcoma is limited. One study loaded APR-246 onto tetrahedral framework nucleic acids (tFNAs), forming the nanocomplex T-APR-246. While APR-246 alone promoted apoptosis and inhibited metastasis in osteosarcoma cells, T-APR-246 demonstrated enhanced stability, efficient delivery, and superior anti-proliferative and anti-metastatic effects, providing a novel rationale for its application [127].

Arsenic Trioxide (ATO), a standard therapy for acute promyelocytic leukemia (APL), has been found to restore mutp53 structure [128]. Crystallographic studies show that arsenic binds to an allosteric site in the p53 core domain, stabilizing its hydrophobic core and rescuing the folding of unstable structural mutants [129]. To enhance targeting and reduce systemic toxicity, researchers developed MDM2-targeting recombinant assembly peptide nanoparticles (MtrapNPs). These nanoparticles encapsulate ATO to restore mutp53 and are surface-modified with p14ARF-mimicking peptides to block MDM2-mediated degradation of renatured p53, showing significant efficacy in osteosarcoma models [130].

ZMC1 (NSC319726) is a novel small-molecule zinc metallochaperone specifically targeting zinc-binding deficient p53 mutants like R175H—a common mutation in osteosarcoma [131]. Wild-type p53 requires zinc for proper folding and stability. The R175H mutation reduces p53’s affinity for Zn^2+^, leading to zinc loss, misfolding, and loss of function [10]. ZMC1 acts as an ionophore, transporting extracellular zinc into the cytoplasm to elevate intracellular zinc concentrations and reactivate mutp53 [132]. Additionally, ZMC1 can chelate redox-active metal ions to increase reactive oxygen species (ROS), inducing p53 post-translational modifications and activating downstream targets [120]. Although still in preclinical exploration, ZMC1’s unique mechanism holds promise as a precision medicine strategy for *TP53*-R175H mutant osteosarcoma.

In summary, therapeutic exploration of the MDM2-p53 pathway has evolved from proof-of-concept to precision targeting. The Nutlins validated MDM2 targeting but were limited by metabolic instability. Subsequent agents like RG7112 and RG7388 improved potency but faced clinical setbacks due to toxicity (e.g., myelosuppression). In contrast, next-generation inhibitors like AMG-232 and APG-115 show promising clinical potential, with AMG-232 demonstrating tolerability in Phase I trials and APG-115 showing anti-tumor activity in Phase II studies. However, monotherapy strategies universally face challenges, as single-pathway blockade often fails to overcome osteosarcoma’s high heterogeneity. Emerging evidence of MDM2 inhibitors’ synergy with chemotherapy and immunotherapy is now driving a paradigm shift toward combination therapies.

### 4.2. Combination Therapy Strategies

To overcome monotherapy toxicity and resistance, combination therapies have become a mainstream direction in osteosarcoma clinical research, primarily focusing on chemosensitization, immune synergy, and targeted therapy combinations.

Chemotherapeutic agents like doxorubicin and cisplatin induce DNA damage, activating ATM/ATR kinases that phosphorylate and stabilize p53. Combining these agents with MDM2 inhibitors concurrently activates p53 and inhibits its degradation, facilitating p53 nuclear accumulation and enhancing anti-tumor effects [133]. For instance, in multidrug-resistant (MDR) osteosarcoma cells, the MDM2 inhibitor NSC59984 combined with doxorubicin significantly enhanced doxorubicin’s activity and DNA damage-induced apoptosis [134].

In immunotherapy, research reveals that MDM2 inhibitors activate p53 while also inducing feedback upregulation of PD-L1 on tumor cells. Thus, combining MDM2 inhibitors with PD-1/PD-L1 inhibitors can elicit synergistic immune responses [135]. Beyond APG-115, AMG-232 has been shown to increase intratumoral CD8+ T cell infiltration. In PD-1 inhibitor-resistant patients, AMG-232 restores p53 function and induces immunogenic cell death (ICD), releasing tumor antigens to reactivate anti-tumor immunity [136].

Combining targeted therapies can also yield synergistic effects. For example, the *MDM2* and *CDK4* genes are co-localized on chromosome 12q13-15. Exploiting this, combining MDM2 inhibitors (e.g., DS-3032b, BI 907828) with CDK4/6 inhibitors (e.g., palbociclib, ribociclib) produces synergistic anti-tumor effects, enhancing cell cycle arrest, promoting apoptosis, and attenuating drug resistance [137].

### 4.3. Innovative Therapies

Despite advances with novel targeted agents, conventional therapies remain limited against recurrence, metastasis, and chemoresistance. Recent innovations have introduced novel therapeutic modalities.

#### 4.3.1. Gene Therapy

Recombinant human p53 adenovirus injection (Gendicine), approved in China in 2003 for head and neck squamous cell carcinoma (HNSCC), is the world’s first marketed gene therapy. It uses a replication-defective adenovirus serotype 5 (Ad5) vector to deliver wild-type *TP53* into target cell nuclei, leading to high p53 expression that triggers cell cycle arrest, apoptosis, and senescence [138]. In osteosarcoma, Gendicine is often combined with chemotherapy or radiotherapy, showing superior anti-tumor effects and improved survival rates compared to monotherapy [139]. With advanced viral vector development, p53-based gene therapy holds promise for broader clinical application.

#### 4.3.2. PROTACs

**Figure 6:**
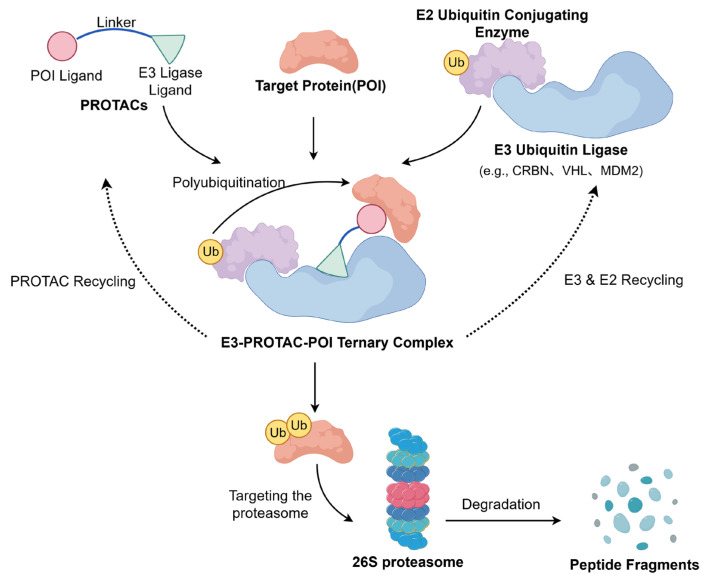
Mechanism of PROTAC-mediated target protein degradation. PROTACs are bifunctional molecules that hijack the intracellular ubiquitin-proteasome system (UPS) to specifically knock down a protein of interest (POI). In the catalytic degradation cycle, the PROTAC molecule initially recruits both the POI and an E3 ubiquitin ligase via its specific ligands, forming a stable “E3-PROTAC-POI” ternary complex. Subsequently, with the assistance of an E2 ubiquitin-conjugating enzyme, the E3 ligase transfers ubiquitin (Ub) to the target protein, initiating its polyubiquitination. Following this, the polyubiquitinated target protein is specifically recognized by the 26S proteasome, translocated to the 20S catalytic core, and degraded into peptide fragments. Ultimately, upon POI degradation, the intact PROTAC molecule is released from the complex and recycled to induce subsequent rounds of degradation. (Created by Figdraw, accessed on 26 February 2026).

Proteolysis-targeting chimeras (PROTACs) represent an innovative strategy to restore p53 activity. The mechanism by which these molecules operate is illustrated in Figure 6. PROTACs are bifunctional molecules comprising an MDM2-binding ligand linked to an E3 ubiquitin ligase ligand. Unlike inhibitors, PROTACs induce complete degradation of the MDM2 protein, eliminating all its oncogenic functions [140]. For example, researchers conjugated the MDM2 inhibitor RG7112 with VH032 (a VHL E3 ligase ligand) to create the PROTAC molecule YX-02-030. This molecule triggers proteasome-mediated MDM2 degradation, effectively activating wild-type p53 and promoting degradation even in p53-deleted or mutant cells [141].

#### 4.3.3. Nanomedicine

Nanoparticles offer significant advantages as drug delivery systems due to their excellent biocompatibility, enabling reduced drug doses and enhanced therapeutic efficacy [142]. Nanotechnology addresses challenges associated with MDM2 inhibitors and p53 reactivators, such as poor solubility, high toxicity, and weak targeting. For instance, lipid nanoparticles (LNPs) have been used to deliver mRNA encoding wild-type *p53* into tumor cells for direct expression of functional p53 protein [143]. Another study in non-small cell lung cancer developed the DP3-p53 formulation, which induced G1 arrest and apoptosis in tumor cells. Administered intratracheally, it significantly inhibited orthotopic lung tumor formation and extended survival in mouse models [144].

## 5. Translational Challenges and Future Directions of Targeting the MDM2-p53 Pathway in Osteosarcoma

As the most common primary malignant bone tumor in children and adolescents, osteosarcoma remains associated with unsatisfactory outcomes despite multimodal therapy, with overall survival rates having stagnated for patients with metastatic or recurrent disease. While neoadjuvant chemotherapy has improved outcomes in localized osteosarcoma, the prognosis for patients with metastasis or chemotherapy resistance remains poor. With advances in understanding the MDM2-p53 pathway, multiple therapeutic agents targeting this axis have shown promise in preclinical studies. However, their clinical translation has been hindered by significant toxicities and the emergence of resistance mechanisms.

### 5.1. Resistance Mechanisms and Challenges

**Figure 7:**
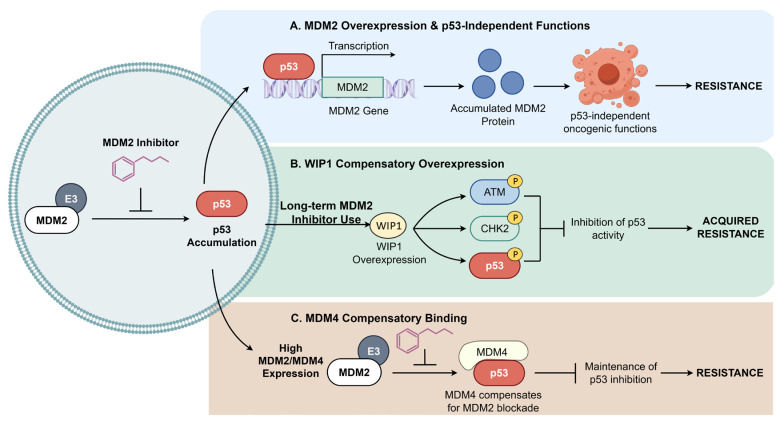
Mechanisms of acquired resistance to MDM2 inhibitors in osteosarcoma. This figure illustrates three major feedback loops that limit the long-term efficacy of MDM2 inhibitors. (**A**) MDM2 feedback accumulation and p53-independent functions: Treatment with MDM2 inhibitors elevates p53 levels; however, p53, functioning as a transcription factor, concurrently induces substantial expression of the *MDM2* gene. The accumulated MDM2 protein, reaching supra-physiological levels, interacts with proteins such as E2F1 and HIF-1α exerting oncogenic effects via p53-independent pathways to drive tumor progression. (**B**) WIP1-mediated dephosphorylation: Long-term administration of MDM2 inhibitors induces compensatory overexpression of the phosphatase *WIP1*. WIP1 dephosphorylates p53 and its upstream activators (ATM and CHK2), thereby reducing p53 protein expression levels and significantly inhibiting its transcriptional activity, which confers acquired resistance to the tumor. (**C**) MDM4 compensatory inhibition: MDM4 is a structural homolog of MDM2. Under conditions where MDM2 is blocked, MDM4 compensatorily binds to and inhibits p53, leading to the emergence of drug resistance. (Created by Figdraw, accessed on 26 February 2026).

Acquired resistance is a major obstacle in osteosarcoma treatment. Beyond *TP53* mutations, compensatory feedback mechanisms involving MDM2 play a key role. Three major feedback loops responsible for this acquired resistance are delineated in Figure 7. First, as shown in Figure 7A, since p53 transcriptionally activates MDM2, pharmacological MDM2 inhibition (e.g., Nutlins) elevates p53 levels, which in turn drives massive MDM2 expression [145]. Although MDM2 inhibitors block the p53-MDM2 interface, the accumulated MDM2 protein can still exert p53-independent oncogenic effects by interacting with other partners like E2F1 and HIF-1α, enabling tumor cell survival [146]. Second, Figure 7B outlines the compensatory overexpression of the phosphatase *WIP1* induced by prolonged MDM2 inhibition. This phosphatase dephosphorylates p53 and its upstream kinases (ATM, CHK2), reducing p53 stability and transcriptional activity, thereby fostering resistance [147]. Furthermore, Figure 7C illustrates the compensatory binding of MDM4. As a structural homolog of MDM2, MDM4 can also inhibit p53. Early-generation MDM2 inhibitors exhibit low affinity for MDM4. In tumors with high MDM2 and MDM4 co-expression, inhibiting MDM2 alone allows MDM4 to maintain p53 suppression, leading to therapeutic escape [148]. Therefore, disrupting these feedback loops is essential for durable efficacy.

### 5.2. Targeted Therapy-Related Toxicity

The most significant translational hurdle for MDM2 inhibitors is on-target toxicity, particularly myelosuppression. Under physiological conditions, p53 activity is tightly regulated; MDM2 inhibition leads to widespread p53 activation, triggering cell cycle arrest and apoptosis via the Bax/Bak-mediated mitochondrial pathway in sensitive tissues. This affects platelets, leukocytes, and megakaryocytes, causing severe hematological toxicity [149]. This dose-limiting toxicity often prevents the attainment of intratumoral drug concentrations sufficient for robust antitumor efficacy. Future strategies should focus on improving therapeutic windows, such as developing tumor-selective delivery systems (e.g., bone-targeted nanocarriers) or optimizing dosing regimens (e.g., intermittent schedules based on pharmacokinetics) to mitigate myelosuppression while maintaining efficacy [150].

### 5.3. Biomarker-Based Precision Medicine

Osteosarcoma exhibits profound intratumoral heterogeneity. Relying solely on static biomarkers like *TP53* mutation or *MDM2* amplification fails to capture the dynamic molecular landscape. Advances in high-throughput sequencing, proteomics, and liquid biopsy are shifting biomarker discovery toward multi-dimensional molecular profiling. Liquid biopsy analytes—such as circulating tumor DNA (ctDNA), exosomes, and non-coding RNAs—offer high sensitivity for early detection, minimal residual disease (MRD) monitoring, and prognostic assessment, addressing the limitations of conventional imaging [151,152]. Concurrently, single-cell and spatial transcriptomics are elucidating the complex tumor microenvironment (TME), providing insights into therapy resistance and enabling personalized treatment strategies [153].

Future biomarker development will emphasize dynamic monitoring and integration of multi-modal data. Combining genomics, clinical pathology, and radiomics via artificial intelligence can enhance diagnostic accuracy and therapeutic personalization [154]. Furthermore, multi-omics frameworks integrating genomic, transcriptomic, and epigenomic data, complemented by spatial omics to resolve tumor heterogeneity, will facilitate the identification of novel targets and improve long-term outcomes [155,156].

### 5.4. Novel Combination Therapy Strategies and Emerging Technologies

Emerging technologies are opening new avenues for targeting the MDM2-p53 axis beyond traditional small molecules. First, single-cell RNA sequencing (scRNA-seq) is dissecting the cellular composition and immune landscape of the osteosarcoma TME, providing a foundation for precision medicine and novel drug discovery [157]. Second, PROTACs offer a paradigm-shifting strategy by recruiting E3 ubiquitin ligases to degrade MDM2 protein completely, potentially overcoming resistance driven by MDM2 feedback accumulation [158]. Additionally, computational approaches like AlphaFold are revolutionizing drug design. By accurately predicting the conformations of peptide-MDM2 complexes, AlphaFold enables the de novo design of cyclic peptides tailored to the MDM2 binding pocket, establishing a new computational paradigm for developing high-affinity, cell-permeable inhibitors [159]. Leveraging such deep learning algorithms will accelerate the development of next-generation MDM2-p53 inhibitors with improved specificity and efficacy [160].

## 6. Conclusions

The MDM2-p53 axis serves as the pivotal driver of osteosarcoma initiation, progression, and chemoresistance. In this malignancy, frequent amplification of the *MDM2* gene and subsequent ubiquitin-mediated degradation of p53 represent the predominant mechanisms for inactivating wild-type p53. Consequently, restoring p53 tumor-suppressive function has emerged as a highly promising strategy in precision oncology. Significant progress has been made, from the discovery of classic inhibitors like Nutlins to the clinical evaluation of next-generation agents such as Milademetan and APG-115. However, severe hematological toxicities observed in early clinical feedback and acquired resistance driven by feedback upregulation of MDM2 proteins remain major obstacles to clinical translation.

Looking forward, the therapeutic landscape is shifting from single-agent antagonism toward a multi-dimensional integration of technologies. Future strategies will likely encompass: (1) employing PROTAC technology to directly degrade MDM2 proteins, thereby circumventing non-canonical oncogenic effects associated with protein accumulation; (2) utilizing bone-targeted nanodelivery systems and intermittent dosing regimens to mitigate systemic toxicity; and (3) leveraging single-cell sequencing and AI algorithms to decipher osteosarcoma heterogeneity and optimize combination strategies. This convergence of multi-dimensional approaches holds the potential to unlock the therapeutic promise of the MDM2-p53 pathway, offering a viable and scientifically grounded path toward personalized recovery for patients with osteosarcoma.

## Figures and Tables

**Table 1:** Small-Molecule Inhibitors Targeting MDM2.

Drug Name	Chemical Structure	IC_50_	Mechanism & Physiological Effects	Clinical Stage	Clinical Research Progress	References
Nutlin-3a	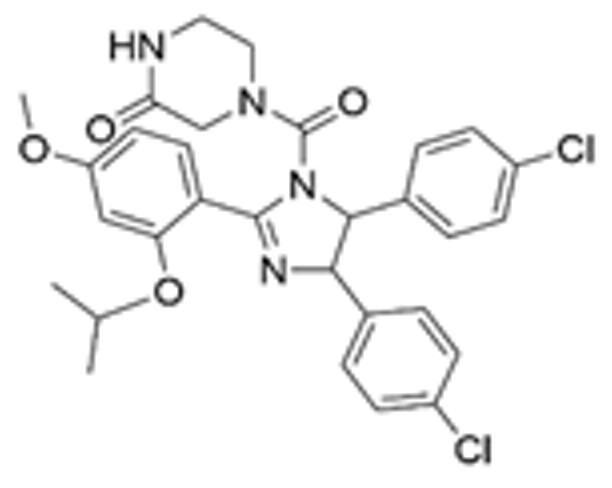	0.09 µM	Binds to MDM2 and blocks its interaction with p53, leading to p53 stabilization, p21 upregulation, and induction of G_1_/S and G_2_/M cell cycle arrest and apoptosis.	Preclinical	Serves primarily as a preclinical tool compound to validate the MDM2-p53 interaction mechanism; has not advanced to large-scale human clinical trials.	[17,79,80]
RG7112	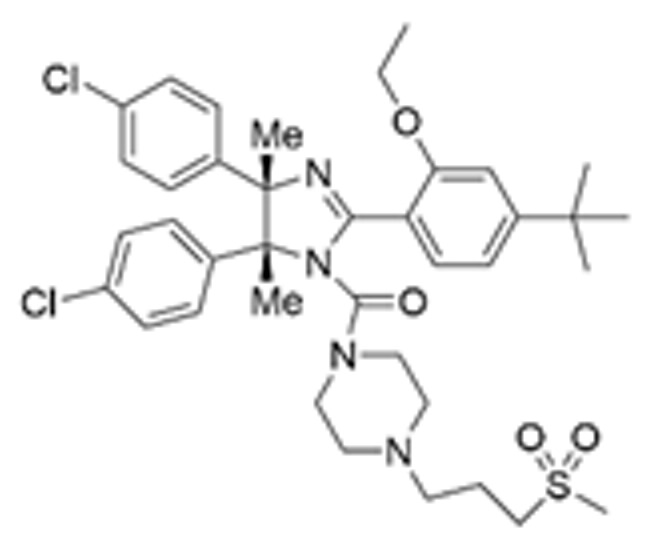	0.018 µM	Activates the p53 pathway, inducing G_1_/S phase cell cycle arrest and apoptosis.	Phase I (Terminated)	No osteosarcoma-specific clinical trials were conducted. In a study involving 116 patients with hematologic malignancies, RG7112 demonstrated limited efficacy and was associated with severe thrombocytopenia and neutropenia. Development shifted toward next-generation agents with improved toxicity profiles.	[81,82]
Idasanutlin (RG7388)	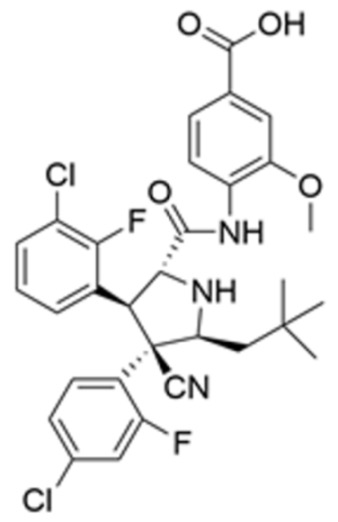	6 nM	Induces robust p53 activation, resulting in G1 and G2/M cell cycle arrest alongside apoptosis.	Phase I/II (Terminated)	In trials for pediatric/adolescent solid tumors (including osteosarcoma), monotherapy failed to elicit objective responses and caused significant myelosuppression and gastrointestinal toxicity. Global development was terminated in 2024.	[83,84]
AMG-232 (Navtemadlin)	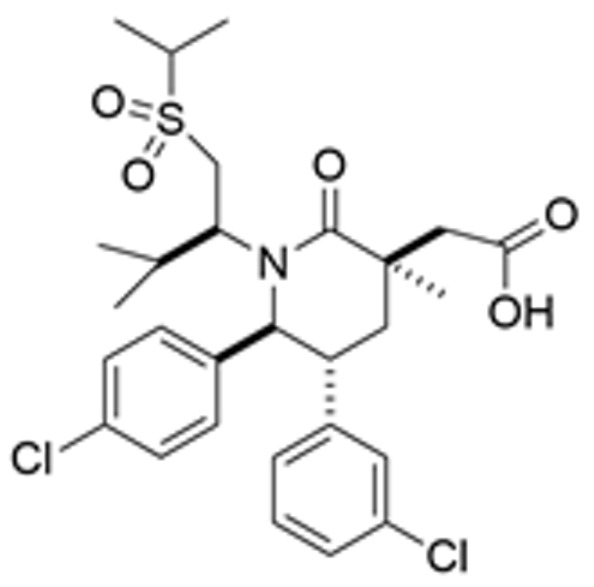	9.1 nM	Activates p53 and significantly upregulates p21, inducing dual G_1_ and G_2_/M cell cycle arrest and robust apoptosis.	Phase I (Advanced Solid Tumors)	In a Phase I clinical trial, AMG-232 demonstrated good tolerability in various advanced *TP53* wild-type solid tumors and multiple myeloma. Current efforts have shifted toward studies in wild-type p53 endometrial cancer and combinations with JAK inhibitors.	[85,86]
Milademetan (DS-3032b)	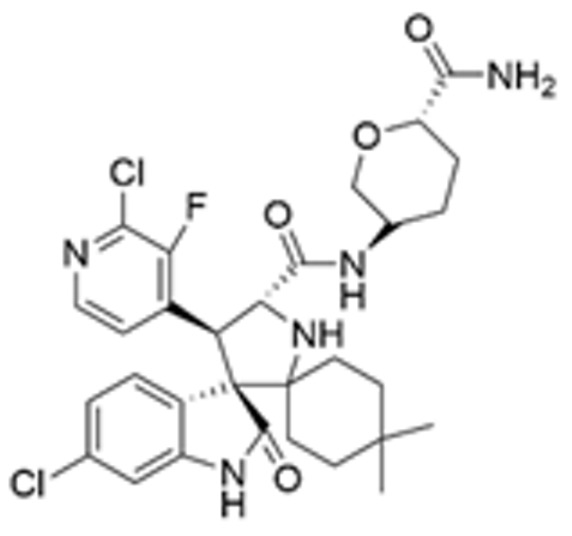	5.57 nM	Stabilizes wild-type p53 and induces strong expression of downstream target genes, triggering G_1_ phase cell cycle arrest, apoptosis, and cellular senescence.	Phase II	In trials for MDM2-amplified advanced solid tumors, despite demonstrating modest activity, the duration of remission was short. Current research is exploring intermittent dosing strategies to mitigate myelosuppression.	[87,88,89,90]
APG-115 (Aliezuhuma)	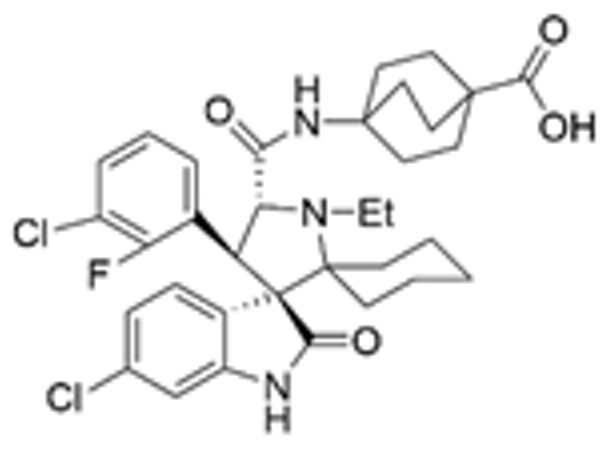	3.8 ± 1.1 nM	Upregulates p53 and p21, primarily inducing G_0_/G_1_ cell cycle arrest and apoptosis. Also activates CD4^+^ T cells to enhance anti-tumor immunity.	Phase II	Demonstrated a favorable safety profile and anti-tumor activity in advanced solid tumors, particularly those that are *TP53* wild-type and *MDM2*-amplified. Combination strategies with immunotherapy and targeted therapy are actively advancing, promising to provide new treatment options for patients currently lacking effective choices.	[91,92]
SAR405838 (MI-77301)	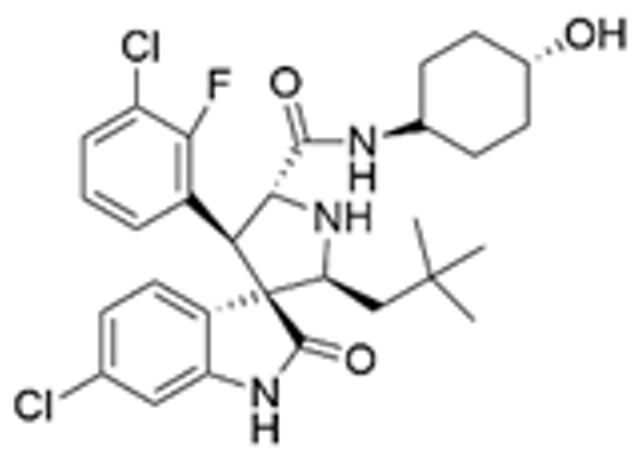	92 nM	Stabilizes and activates the p53 pathway, inducing apoptosis, G_1_/S or G_2_/M cell cycle arrest, and inhibition of cell proliferation.	Phase I	In studies targeting dedifferentiated liposarcoma, SAR405838 showed preliminary safety in patients with specific molecular subtypes, but monotherapy efficacy remains to be improved.	[93,94,95]
HDM201 (Siremadlin)	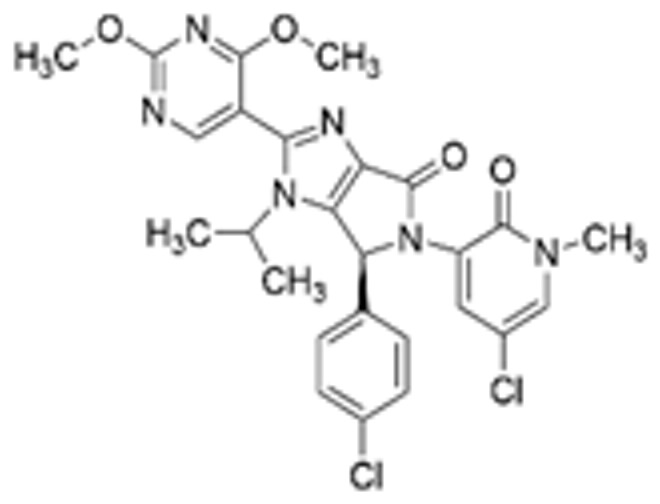	0.13 nM	Activates the p53 signaling axis, inducing potent p53-dependent cell cycle arrest and apoptosis.	Phase II	In the ADORE trial, siremadlin combined with ruxolitinib for myelofibrosis demonstrated a reduction in spleen volume but was associated with adverse events such as anemia and thrombocytopenia. It is currently being investigated as a maintenance therapy following hematopoietic stem cell transplantation.	[96,97]
Sulanemadlin (ALRN-6924)	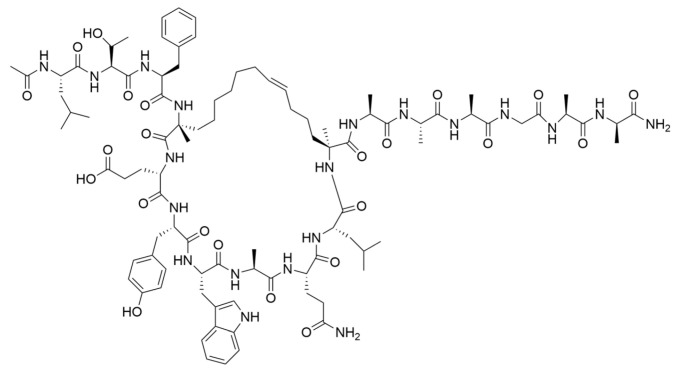	0.2–3.3 µM	Dual MDM2/MDM4 inhibitor that restores p53-mediated apoptosis and induces reversible cell cycle arrest, designed to minimize myelosuppressive toxicity.	Phase I	In a clinical trial (https://clinicaltrials.gov/study/NCT05622058, accessed on 26 February 2026) designed to evaluate ALRN-6924 for protecting patients with *TP53*-mutant breast cancer from chemotherapy-induced toxicity, the combination of this agent with adjuvant chemotherapy resulted in severe hematological adverse events. Consequently, its clinical development as a chemoprotectant has been terminated.	[98]

Note: Chemical structures in this table were generated using ChemDraw (Version: 23.1.1.3).

**Table 2:** Compounds Targeting Mutant p53.

Drug Name	Chemical Structure	IC_50_	Mechanism & Physiological Effects	Clinical Stage	Clinical Research Progress	References
APR-246	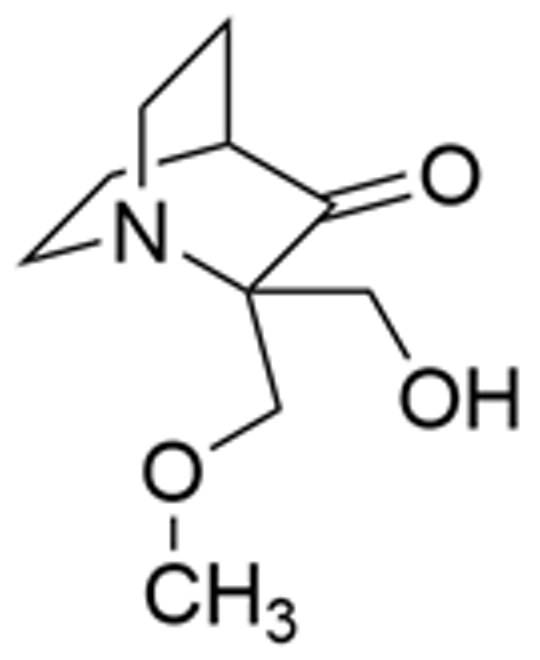	8.8 ± 0.1 μM	Induces conformational refolding of mutant p53, triggering profound apoptosis and cell cycle arrest, while simultaneously depleting intracellular glutathione (GSH) to cause massive reactive oxygen species (ROS) accumulation and induce ferroptosis.	Phase II	In studies treating high-risk MDS/AML patients with *TP53* mutations, APR-246 combined with azacitidine demonstrated a high complete remission rate and safety.	[113,114,115]
Arsenic Trioxide (ATO)	As_2_O_3_	0.05–2.4 µM	Binds to a cryptic allosteric site of mutant p53 and restores its transcriptional activity, inducing p53-dependent apoptosis and dual G1/G2 phase cell cycle arrest.	Phase I/II	No sarcoma-related clinical trials have been conducted yet. In tumors with high-frequency *TP53* mutations, such as Triple-Negative Breast Cancer (TNBC), ATO demonstrates potent p53 reactivation capability and favorable anti-tumor potential.	[116,117]
ZMC1 (NSC319726)	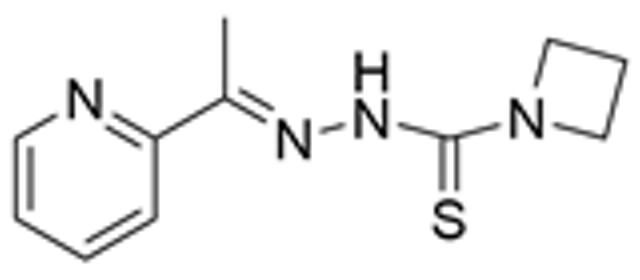	8 nM	Specifically restores zinc-deficient p53 mutants to rapidly trigger profound apoptosis; additionally, chelates redox-active metal ions to elevate intracellular reactive oxygen species (ROS) levels.	Preclinical	Remains in the preclinical stage. Studies have elucidated the optimal concentration for ZMC1 to restore p53 conformation; current research focuses on screening patient populations suitable for entry into first-in-human trials.	[118,119,120]
COTi-2	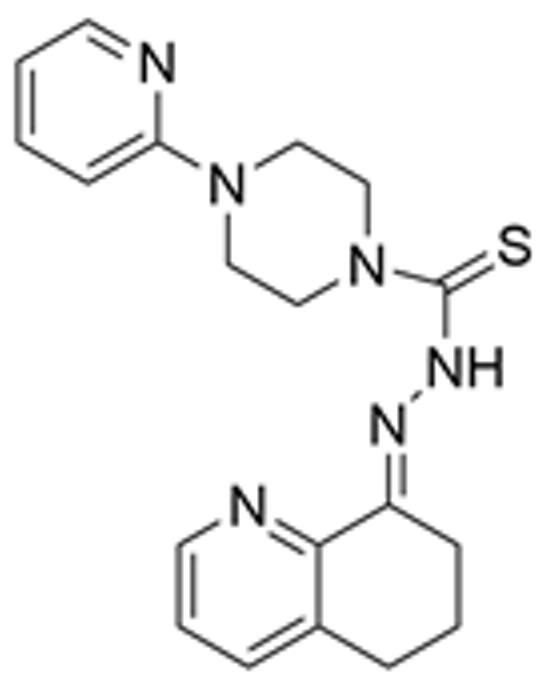	1.4–13.2 nM	Metallodrug restoring mutant p53 conformation to trigger apoptosis and cell cycle arrest.	Phase I/II	Currently in Phase I/II clinical trial (https://clinicaltrials.gov/study/NCT02433626, accessed on 26 February 2026), aiming to evaluate its safety and preliminary efficacy in advanced solid tumors.	[121,122]
PK11007	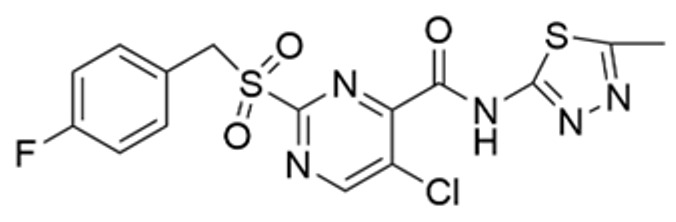	2.3 μM	Specifically alkylates cysteine residues on mutant p53 to stabilize its conformation, leading to robust transcription of p53 target genes and subsequently triggering cell cycle arrest and potent apoptosis.	Preclinical	Currently under in-depth preclinical investigation. PK11007 possesses good anti-tumor potential due to its ability to correct the conformation of mutation hotspots such as Y220C and induce cellular oxidative stress.	[123,124,125]

Note: Chemical structures in this table were generated using ChemDraw (Version: 23.1.1.3).

## Data Availability

No new data were created or analyzed in this study. Data sharing is not applicable.

## Abbreviations

The following abbreviations are used in this manuscript:

AI	Artificial Intelligence
AML	Acute Myeloid Leukemia
ATO	Arsenic Trioxide
CDK	Cyclin-Dependent Kinase
ctDNA	Circulating Tumor DNA
CTD	C-terminal Regulatory Domain
DBD	DNA-Binding Domain
EMT	Epithelial–Mesenchymal Transition
GOF	Gain-of-Function
HNSCC	Head and Neck Squamous Cell Carcinoma
IC_50_	Half-maximal Inhibitory Concentration
ICD	Immunogenic Cell Death
LFS	Li-Fraumeni Syndrome
LNP	Lipid Nanoparticles
lncRNA	Long Non-coding RNA
MARs	Matrix Attachment Regions
MDM2	Murine Double Minute 2
MDM4	Murine Double Minute 4
MDS	Myelodysplastic Syndromes
miRNA	MicroRNA
MRD	Minimal Residual Disease
mutp53	Mutant p53
ncRNA	Non-coding RNA
NES	Nuclear Export Signal
NLS	Nuclear Localization Signal
OD	Oligomerization Domain
OS	Osteosarcoma
p53	Tumor Protein p53
PRD	Proline-Rich Domain
PROTACs	Proteolysis Targeting Chimeras
ROS	Reactive Oxygen Species
scRNA-seq	Single-cell RNA Sequencing
SNP	Single Nucleotide Polymorphism
TAD	Transactivation Domain
tFNAs	Tetrahedral Framework Nucleic Acids
TME	Tumor Microenvironment
TP53	Tumor Protein p53 Gene
WIP1	Wild-type p53-Induced Phosphatase 1
